# The Impact of Motor Disability and the Level of Fatigue on Adherence to Therapeutic Recommendations in Patients with Multiple Sclerosis Treated with Immunomodulation

**DOI:** 10.7150/ijms.61964

**Published:** 2021-08-27

**Authors:** Robert Ślusarz, Joanna Olkiewicz, Robert Bonek, Karolina Filipska, Monika Biercewicz, Adam Wiśniewski

**Affiliations:** 1 Neurological and Neurosurgical Nursing Department, Faculty of Health Science, Collegium Medicum, Nicolaus Copernicus University, Torun, Poland; 2 Department of Neurology and Clinical Neuroimmunology, Regional Specialist Hospital, Grudziadz, Poland; 3 Clinic of Geriatrics, Faculty of Health Science, Collegium Medicum, Nicolaus Copernicus University, Torun, Poland; 4 Department of Neurology, Faculty of Medicine, Collegium Medicum, Nicolaus Copernicus University, Torun, Poland; 5 Polish Association of Neuroscience Nursing, Poland

**Keywords:** adherence, motor disability, fatigue, multiple sclerosis

## Abstract

**Aim:** The aim of the study was to clarify whether the motor disability and the fatigue-related syndrome affect the level of compliance with therapeutic recommendations.

**Methods:** Prospective studies were conducted among 165 patients treated under the drug program - Treatment of Multiple Sclerosis (MS) at the Department of Neurology and Clinical Neuroimmunology of the Regional Specialist Hospital in Grudziadz (Poland). The research was carried out by the method of diagnostic survey, questionnaire technique with the use of standardized research tools. The Adherence in Chronic Diseases Scale (ACDS) was used to assess the level of compliance with therapeutic recommendations. The Expanded Disability Status Scale (EDSS) was used to assess the degree of disability, and the Modified Fatigue Impact Scale (MFIS) was used to assess the degree of disability. The Chi-square test, Shapiro-Wilk test and Kruskal-Wallis were used.

**Results:** The statistical analysis showed that there is a relationship (p=0.0055) between the patient's motor disability assessed in the EDSS scale and the level of compliance with therapeutic recommendations assessed in the ACDS scale. The higher the patient's disability level (EDSS 4.5-6.5), the lower the treatment adherence rate. The conducted research shows that the average score in the MFIS scale for individual levels of compliance with therapeutic recommendations expressed in the ACDS scale is, respectively: for the low level - 38.3 MFIS points, for the medium level - 34.4 MFIS points and for the high level- 33.2 MFIS points. The obtained results were not statistically significant (p=0.6098).

**Conclusion:** It was found that the level of adherence to therapeutic recommendations in patients with relapsing-remitting multiple sclerosis treated with immunomodulation in the study group remained high. There is a relationship between the patient's disability and the level of adherence to therapeutic recommendations.

## Introduction

Multiple Sclerosis (MS) is a chronic, inflammatory autoimmune disease of the central nervous system (CNS), in the course of which there is disseminated damage to CNS 1-4. Multifocal brain injury is characterized by a wide variety of symptoms, such as paresis, sensory disorders or balance disorders, often accompanied by mental and cognitive disorders or excessive fatigue 5,6. The disease mainly affects young adults aged 20-40 years and is the main cause of motor disability in this age group 7. In addition to motor disability clinical symptomatology also includes fatigue, which is one of the most common and disabling symptoms in multiple sclerosis, disrupting patients' daily lives. The variety of problems in patients with multiple sclerosis is due to the “rich” clinical symptomatology, the progression of the disease itself, as well as the proposed treatment. The most important goal of treatment in MS is to improve the quality of life of patients by reducing the frequency of relapses and preventing the progression of disability. Therefore, the key issue in the management of the patients is compliance by the patient with therapeutic recommendations.

Many well-documented studies conducted around the world present the clinical symptoms and course of MS 1,6,8, the quality of life of patients with MS 9-11, the impact of specific treatment on the functioning of patients 12-14, while there are no reports on the occurrence of motor disability or fatigue-related syndrome in the context of patient compliance with therapeutic recommendations.

Studies conducted around the world indicate that patients do not adhere to therapeutic recommendations mainly due to side effects and ineffectiveness of treatment, which in turn, leads to a deterioration of control over the course of the disease. O´Rourke et al. 15 reported that during the first year of treatment with interferons β 1b and β 1a, drug tolerance was the decisive factor in maintaining the therapy. Similarly, in the studies conducted by Treadaway et al. 16, it was reported that patients who stop treatment are three times more likely to be uncertain about the effectiveness of their treatment. The most common reason for missing a dose was that the patient had forgotten to take it.

Despite significant progress in MS research, the disease still causes many diagnostic difficulties. This is mainly due to the fact that MS is a disease with a very varied course. Adherence to treatment principles is a very important element of the treatment of MS patients and requires cooperation, a positive emotional state and acceptance of the disease 17-19.

The aim of the study was to clarify whether the motor disability and the fatigue-related syndrome affect the level of compliance with therapeutic recommendations.

## Methods

### Study design

This prospective study enrolled 165 patients treated under the drug program - Treatment of Multiple Sclerosis in the Department of Neurology and Clinical Neuroimmunology of the Regional Specialist Hospital in Grudziadz (Poland).

The inclusion criterion for the study was the diagnosis of relapsing-remitting multiple sclerosis (RRMS) diagnosed by a neurologist in accordance with the revised McDonald criteria 20,21 and qualification for immunomodulation treatment under the drug program. Patients with severe depression who did not respond to treatment, diagnosis of epilepsy, or hypersensitivity to any drug used in the program were excluded.

As part of the drug program, an initial meeting was held with patients educate them about the type of therapy they were receiving and associated management issues such as their ability to designate puncture sites, use of the auto-injector, use of the Pen for injection, care of the puncture site, rotation of puncture sites, possible side effects associated with the drug therapy, methods of dealing and minimizing side effects, and adhering to the principles of asepsis and antisepsis. During the next meeting (not shorter than 60 days from qualifying for the program), the patient was assessed for the degree of disability, the level of fatigue, and the level of compliance with therapeutic recommendations.

### Participants

A total of 165 participants were included in this study. Among the surveyed patients, 124 (75.2%) were women and 41 were men (24.8%). The age of the respondents ranged between 18-66 years. Patients aged 21-40 accounted for more than half (86 people - 52.1%). The largest group of respondents was represented by people with higher education - 88 people (53.3%). The vast majority of respondents lived in the city (114 people - 69.1%). The largest group of patients (52 people - 31.5%) were people whose disease duration was 3-5 years. The smallest group were people with a diagnosis in the range of 0-2 years (25 people- 15.1%). Most respondents, (104 [63.0%]) received drugs in the form of subcutaneous or intramuscular injections. The exact characteristics of the study group are presented in Table 1.

### Instruments

The Adherence in Chronic Diseases Scale - (ACDS) 22 was used to assess the level of adherence to therapeutic recommendations. This scale is used to examine the degree of implementation of the therapeutic plan by patients with chronic disease. The scale consists of 7 questions with proposed sets of 5 answers to each question. Each question is scored from 0 (lack adherence) to 4 (high adherence) points. The questions relate to the behaviours that directly determine adherence and to situations and views that may indirectly affect adherence. This tool not only reflects the actual implementation of the therapeutic plan in the field of pharmacotherapy, but also points to the mechanisms that determine patient adherence. According to the authors of the tool, the psychometric properties are satisfactory and the Cronbach's α coefficient is 0.739 22,23. For purposes of statistical analyses, a division of patients was proposed according to the established criteria: score ≤20 -low adherence level, 21-26 -medium level and ≥27 - high adherence level 22.

The Expanded Disability Status Scale (EDSS) was used to assess the degree of disability 24-26.

The Fatigue Impact Scale 27 and the Modified Fatigue Impact Scale (MFIS) 28 were used to determine the degree/level of the impact of fatigue in the patient. For the purposes of statistical analyses, a point value for the entire scale (MFIS) was calculated, separately for the cognitive subscale (cognitive subscale - F_2) and jointly for the physical and psychosocial subscale (physical and psychosocial subscales - F_1 + F_3) 29.

### Statistical analyses

Statistical analysis was performed using Statistica 13 (StatSoft, USA) under the license of Nicolaus Copernicus University, Poland. For the measurable variables, the arithmetic mean (X) and SD were calculated, and for non-measurable variables, the percentages (%) were calculated. All quantitative variables were tested using the Shapiro-Wilk test to determine the type of distribution. The nonparametric Kruskal-Wallis test was used to compare the results between groups for continuous variables, and the chi-squared test was used for categorical data. For all comparisons, the level of α=0.05 was assumed, and P-values were rounded to four decimal places.

### Ethical considerations

The research was approved by the Bioethics Committee of the Nicolaus Copernicus University in Toruń at the Ludwik Rydygier Collegium Medicumin Bydgoszcz (consent no.: KB 669/2018 and 739/2017). The research was voluntary, free and anonymous. All patients gave their written consent to the study, were informed about its purpose, and about the possibility of withdrawing from participation in the study at any stage.

## Results

In most cases, the study groups showed a high level of compliance with therapeutic recommendations (113 people - 68.5%). Statistical analysis showed a statistically significant (p=0.0001) difference in the distribution of patients at individual levels of the ACDS scale (Table 2).

The influence of motor disability defined in the EDSS scale on the level of compliance with therapeutic recommendations expressed in the ACDS scale is presented in Table 3. The data analysis showed that the higher the level of disability in the patient, the lower the level of compliance with therapeutic recommendations. Patients with EDSS scores 4.5-5.0 and 5.5-6.5 show a low (42.9%) or average (6.6%) level of adherence to treatment as expressed in the ACDS scale. On the other hand, patients with EDSS scores of 0.0-2.0 and 2.5-4.0 in the EDSS scale show a high (95.5%) level of compliance with therapeutic recommendations expressed in the ACDS scale. Statistical analysis confirmed the existence of a relationship between the level of disability and the level of compliance with therapeutic recommendations (p=0.0055).

The research showed (Table 4) that the average score in the MFIS scale for individual levels of compliance with therapeutic recommendations expressed in the ACDS scale is, respectively: for the low level - 38.3 MFIS points, for the medium level- 34.4 MFIS points and for the high level-33.2 MFIS points. Statistical analysis showed no statistically significant differences (p=0.6098) between the analysed levels. In the assessment of the impact of fatigue on the physical and psychological dimensions of patients, it can be observed that patients with a low level of compliance with therapeutic recommendations obtained the highest mean (22.9 MFIS points) compared to patients with medium (20.8 MFIS points) and high (19.8 MFIS points) level of adherence to therapeutic recommendations. This means that patients showing greater fatigue in the physical and psychosocial dimensions (22.9 MFIS points) show a low level of therapeutic adherence (ACDS). However, the difference between the groups was not statistically significant (0.5418). The greatest impact of fatigue in the cognitive dimension can be observed in patients with a low level of compliance with therapeutic recommendations (the mean was 15.4 MFIS points). In the remaining groups, the mean values obtained were lower (respectively: 13.8 and 13.4 MFIS points), indicating a lower impact of fatigue on the level of compliance with therapeutic recommendations. However, the obtained difference between the groups was not statistically significant (0.7797).

Furthermore, the analysis showed that the duration of the disease, the therapy used, the treatment duration and the disease relapses did not have a statistically significant effect on the level of therapeutic adherence (ACDS).

## Discussion

The performed statistical analysis showed that there is a relationship between the patient's mobility impairment assessed in the EDSS scale and the level of compliance with therapeutic recommendations assessed in the ACDS scale. The higher the patient's disability level (EDSS 4.5-6.5), the lower the treatment adherence rate.

The obtained result was confirmed in the study by Rio et al. 30, where the authors showed that the higher degree of disability (EDSS), mainly in the first two years of treatment, is the main cause of treatment discontinuation, which is related to non-compliance with therapeutic recommendations. In the studies by Hao et al. 31, it was also found that patients with moderate and high level of adherence to treatment have significantly better mean results as assessed by the EDSS scale than patients with low level of adherence (EDSS - 4.1 and 4.2 vs 4.8; p<0.05). Studies by Bartolomé-García et al. [32]also showed that patients with higher EDSS disability (1.5-6) discontinued MS treatment earlier than patients assessed using the EDSS scale (0-1). In a study of the Mexican patient population with MS 33, it was found that patients with high levels of adherence to treatment had better EDSS scores at the end of treatment compared to patients with lower levels of adherence (p=0.003). In Turkish studies 34, it was shown that higher disability in the EDSS scale correlates with a lower level of adherence to therapeutic recommendations and a higher rate of treatment discontinuation. The worldwide MSBASIS 35, study also confirmed that the increase in the EDSS scale is related to the level of treatment adherence. Different results were obtained in a study by McKay al. 36 who investigated the causes of non-compliance among Canadian patients. Failure to adhere to therapeutic recommendations was associated with a lower EDSS scale (0-2.5 vs 3.0-5.5).

It was also analysed whether the MFIS may influence the patient's compliance with therapeutic recommendations. As demonstrated by the authors' own research, its influence on the level of adherence to therapeutic recommendations was not found. There are no reports in the available literature on the fatigue-related syndrome and its impact on adherence to therapeutic recommendations. Finally, most studies concern the assessment of the level of fatigue in MS patients, correlation with their quality of life 37-39, or the occurrence of depressive disorders 40-43.

## Conclusions

It was found that the level of adherence to therapeutic recommendations in patients with relapsing-remitting multiple sclerosis treated with immunomodulation in the study group remained high. Furthermore there is a relationship between the patient's disability and the level of adherence to therapeutic recommendations. Patients with a higher disability deficit show a lower level of therapeutic adherence. Finally, there was no significant effect of fatigue on the level of compliance with therapeutic recommendations.

## Figures and Tables

**Table 1:** Characteristics of the studied group

Variable	N=165 (100%)
Gender	
Woman	124 (75.2)
Man	41 (24.8)
Age	
≤20 years	4 (2.4)
21 - 40 years	86 (52.1)
≥41 years	75 (45.5)
Education	
Basic (elementary school - 8 years)	2 (1.2)
Secondary (middle school - 12 years)	75 (45.5)
Higher (studies, 17 years)	88 (53.3)
Place of residence	
City	114 (69.1)
Village	51 (30.9)
Duration of the disease	
0-2 years	25 (15.1)
3-5 years	52 (31.5)
6-10 years	44 (26.7)
˃10 years	44 (26.7)
Applied therapy	
Oral medications only	53 (32.1)
Subcutaneous or intramuscular injection drugs only	104 (63.0)
Intravenous medications only	8 (4.9)

**Table 2:** The level of adherence to ACDS therapeutic recommendations

ACDS	N (%)	P-value
ACDS values		
≤20 - low level	7 (4.2%)	
21-26 - average level	45 (27.3%)	0.0001
≥27 - high level	113 (68.5%)	

Chi-squared test

**Table 3:** Impact of motor disability (EDSS) on therapeutic adherence (ACDS)

EDSS		ACDS		P-value
	Low	Medium	High	
0.0-2.0	3 (42.9%)	15 (33.3%)	51 (45.1%)	
2.5-4.0	1 (14.3%)	27 (60.0%)	57 (50.4%)	0.0055
4.5-5.0	2 (28.6%)	2 (4.4%)	3 (2.7%)	
5.5-6.5	1 (14.3%)	1 (2.2%)	2 (1.8%)	

Chi-squared test

**Table 4:** The impact of fatigue (MFIS) and therapeutic adherence (ACDS)

		ACDS		P-value
	Low	Medium	High	
**MFIS**				0.6098
X ± SD	38.3 ± 25.4	34.4 ± 17.4	33.2 ± 17.9	
Scope	4.0-69.0	3.0-68.0	0.0-79.0	
Median	47.0	38.0	31.0	
**Physical and psychosocial subscales - F_1 + F_3**				0.5418
X ± SD	22.9 ± 16.3	20.8 ± 9.8	19.8 ± 10.6	
Scope	0.0-40.0	2.0-39.0	0.0-43.0	
Median	29.0	22.0	19.0	
**Cognitive subscale - F_2**				0.7797
X ± SD	15.4 ± 9.9	13.8 ± 9.3	13.4 ± 9.3	
Scope	3.0-29.0	0.0-35.0	0.0-36.0	
Median	15.0	14.0	13.0	

Kruskal-Wallis test

**Table 5:** Comparative characteristics of patients in terms of the ACDS scale and medical factors

	Low	Medium	High	*P*-value
**Duration of the disease**				0.0858
0-2 years	2 (28.6%)	1 (2.3%)	22 (19.5%)	
3-5 years	3 (42.9%)	14 (31.8%)	34 (30.1%)	
6-10 years	0 (0.0%)	16 (36.4%)	28 (24.8%)	
>10 years	2 (28.6%)	13 (29.5%)	29 (25.7%)	
**Therapy applied**				0.5918
Oral medications	3 (42.9%)	12 (26.7%)	38 (33.6%)	
Subcutaneous or intramuscular injection drugs	4 (57.1%)	32 (71.1%)	68 (60.2%)	
Intravenous medications	0 (0.0%)	1 (2.2%)	7 (6.2%)	
**Duration of treatment**				0.4300
0-2 years	4 (57.1%)	11 (25.0%)	40 (36.4%)	
3-5 years	1 (14.3%)	17 (38.6%)	33 (30.0%)	
6-10 years	1 (14.3%)	14 (31.8%)	27 (24.5%)	
>10 years	1 (14.3%)	2 (4.5%)	10 (9.1%)	
**Relapses of the disease**				0.0849
Yes	5 (71.4%)	18 (40.0%)	65 (57.5%)	
No	2 (28.6%)	27 (60.0%)	48 (42.5%)	

Chi-square test
